# Neuromarketing in Haute Cuisine Gastronomic Experiences

**DOI:** 10.3389/fpsyg.2020.01772

**Published:** 2020-08-04

**Authors:** Ana Mengual-Recuerda, Victoria Tur-Viñes, David Juárez-Varón

**Affiliations:** ^1^Departamento de Organización de Empresas, Universitat Politècnica de València, Valencia, Spain; ^2^Department of Communication and Social Psychology, University of Alicante, Alicante, Spain; ^3^Instituto de Tecnología de Materiales, Universitat Politècnica de València, Valencia, Spain

**Keywords:** psychology, neuromarketing, haute cuisine, gastronomic experience, experiential marketing, galvanic skin response, electroencephalography, eye tracking

## Abstract

Gastronomic experiences offer a set of stimuli that affect the customer’s perception of chef-designed food. This empirical study aims to analyze the influence on the consumer, at a cerebral level, of the stimuli characteristic of a high-level gastronomic experience, in a Michelin starred restaurant. The presentation by the waiter or chef, the plate design, the dish served, the taste of food, interaction or moment in which the food is served are the variables analyzed. Through the use of neuromarketing techniques – galvanic skin response to register emotional arousal, eye tracking to identify where consumers look, and electroencephalography to interpret emotional reactions – combined with qualitative research technique (In-depth interviews with all consumers), in order to know the natural and suggested memories, the objective of this research is to determine the emotional impact of the variables analyzed against the actual taste of food, obtaining conclusions about each variable in overall experience and allowing the authors to propose a model of order design of dishes, designed by the chef, based on emotions and thereby achieving greater efficiency in results of the experience and the memory of it. Results indicate a favorable influence on emotions when the chef presents the food. Likewise, dishes with special presentation have a greater influence at the level of interest than conventional dishes. It is important to highlight that the levels of emotion and attention fall after the midway point of the experience, due to the duration of the experience. Therefore, the dishes do not have the same emotional impact, despite being as special as at the beginning of the experience.

## Introduction

One of the most traditional sectors of any economy is the restaurant sector (Díaz, 2015). It is a cyclical sector that is in line with the economic situation of the country, so that it grows when the country goes through a favorable economic moment (Ostelea, 2018). The World Tourism Organization (UNWTO, 2017) dependent on the United Nations, indicates that, in the world restaurant scenario, the countries in which the greatest investment is generated are the, China and Japan, with 514,600, 485,600 and 234,500 million Euros, respectively. In the United States alone, the restaurant industry turns over $ 899 billion, with more than 1 million restaurants spread throughout the country (UNRA, 2020). The consumers that spend the most on restaurants are the Japanese, those of the United States and Canadians, with more than € 1,100 per inhabitant, unlike the Germans and the Chinese with an expenditure of € 412 and € 350 per inhabitant, respectively (Díaz, 2015).

Although the perspectives of the restaurant sector are optimistic, this sector must face many challenges derived from new consumer habits (Bertoch, 2018) which are being influenced by factors such as technological changes, the need to try new experiences or the increase in competition in the sector (IFMA, 2017). Continuous innovation is the secret to keeping the restaurant sector attractive, adapting to the trends and needs of new generations of consumers. The current consumer associates restaurants with experience, either because they consider it as an alternative to household chores, or because they can enjoy the moment intensely, sharing with family or friends; or looking to be surprised with new flavors. The challenge for the sector is to offer unique and memorable experiences, making use of culinary creativity, the decoration of the premises and tables, quality of service, special events and technological level.

The culinary movement of haute cuisine, also known as techno-emotional (or molecular) cuisine, has as its main objective to excite the diner (Moreno, 2015). It consists of a gastronomic modality in which you take more risks in the elaboration of the dishes, playing, apart from the textures and temperatures of the food, with the five senses. In this way, the diner remains participatory during the meal, enjoying the fumes, essences, colors and visual effects. It is a type of cuisine that requires time for research, design and preparation and that takes care of even the smallest detail. The establishments must set themselves apart and sell a unique experience to the client, resorting to experiential and sensory marketing. This is the direction to follow to satisfy customer expectations and build loyalty.

Haute cuisine is one of the most important tourist attractions of large cities worldwide, and the most famous chefs and restaurants in the United States, Europe, and Japan have spent years working on their personal brands and making themselves known in the Middle East, China and India. The Michelin guide (Michelin, 2019) whose original mission was to contribute to better mobility, has become a world reference in gastronomy, publishing 28 Michelin Guides in 25 countries. From the year 1926, the valuation of the restaurants with stars began to be incorporated into the guide and it was in 1936 when the valuation criteria applied when awarding the stars were released.

Michelin has 85 inspectors around the world. All of them are anonymous and salaried, professionals with an excellent preparation (instructed in hospitality schools and with a minimum professional experience of 5 years) and follow the same criteria and methodology throughout the world. Subsequently, the organization itself trains them, teaching the criteria for the attribution of the stars, the comfort categories and how other qualities and services are to be valued (they are accompanied by another experienced inspector, for their first 6 months). All inspectors must rigorously follow the same quality evaluation criteria and follow the same standards on their visits. The main rules are: independence, anonymous visit, choice of the best, annual update and homogeneity of the selection. Annually, an inspector has to travel more than 30,000 kilometers to visit some 800 establishments, including 250 meals in restaurants and around 150 nights in hotels.

In the world there are 136 restaurants with three Michelin stars, spread across 17 countries (Cancela, 2019). Currently, Spain is the fifth highest country in the world regarding number of Michelin stars, with a total of 206 stars (11 new stars compared to 2018), maintaining the fifth position, leading Japan in the ranking.

## Materials and Methods

The aim of this research is to determine through neuromarketing techniques the cognitive perception of consumers, between 35 and 55 years old, with a medium-high socio-economic level, who like to experience a gastronomic experience of haute cuisine. The level of learning is linked to the influence of the presentation, the food and the moment in which it is served. The rest of the aspects (restaurant, atmosphere, table and menu) remain constant. To do this, we have used neuromarketing techniques that have allowed us to analyze the attention of the subjects to the stimuli (eye tracking), the emotional intensity experienced (Galvanic Skin Response) and the interpretation of the sensations and emotions experienced (electroencephalography).

### Objectives

This research work helps answer the question of which aspects are more relevant for consumers in haute cuisine gastronomic experiences. The experience was carried out in a restaurant with 2 Michelin stars called L’Escaleta, located in Alicante (Spain), whose chef, Kiko Moya, seeks an identity of his own, without falling into reconstruction, based on the flavors of the region, aiming to contribute something new from the traditional flavors.

The main objective of the research is to analyze the mixed construct type “experience” of a consumer, in response to the presentation (description) and tasting of food in a restaurant.

The specific objectives are as follows:

• Monitor, by synchronizing the galvanic response of the skin, the gaze fixation (pupil trajectory) and the recording of emotional levels (through electroencephalography) to identify the emotional traces (somatic markers) based on the levels of the bio-measurements recorded, for each phase and for the overall experience.
• Measure the perception of value of the tasting experience (based on the measurement of emotional or arousal intensity, and the percentage value of the register of emotions, expressed in terms of “engagement,” “excitement,” “interest,” “focus,” “Stress,” and “relaxation”).
• Register areas and elements of greatest interest to the consumer and verify that they coincide with the elements that add value to the product (design of the dish or support and presence of the Chef) and are manifested based on the increase in emotional arousal and the degree of interest by the dish.
• Analyze whether the perception of value of the sensory experience can be improved by modifying the order of the dishes, based on the results of the registered biomedical measurements.

### Research Instrument

In this study, the research technique used is neuromarketing. Its purpose is to measure the cognitive processing of the stimuli designed in a haute cuisine gastronomic experience. Neuromarketing combines neuroscience, psychology and economics (Madan, 2010) analyzing the effectiveness of brand stimuli (Baron et al., 2017) and the psychology of consumer behavior (Plassmann et al., 2012) improving conventional research methods, limited by participants’ perceptions or behaviors (Ariely and Berns, 2010).

Eye tracking, galvanic skin response (GSR) and electroencephalography (EEG) are the three specific neuromarketing techniques used in this work. Eye tracking has been used to record the visual attention of the subjects based on their eye movements (Duchowski, 2007) identifying the areas that are of interest to the subject (AOI). The GSR collects electrodermal activity (EDA), reflecting changes in the state of emotional arousal, influencing the cognitive perception of stimuli (Critchley, 2002). The EEG provides valuable information on brain activity, analyzing and recording changes in electrical currents, in the form of brain waves (Yadava et al., 2017). When subjects focus their attention on a stimulus, it is recorded by the eye tracking system and cognitive and affective processing (partially recorded by GSR and EEG) begins, resulting in an influence on consumer preferences (Bornstein and D’agostino, 1992; Ramele et al., 2012; Sanei and Chambers, 2013).

Market research has not had the impact it should have on business decision making (Martínez, 1991). The outputs generated by the market research department should have a direct impact on the economic results of companies (Báez and De Tudela, 2007), however, between 75 and 85% of all new product launches fail (Innerscope, 2018) often due to failing to predict major changes and new trends. Neoclassical economics, specifically the concept of the Homo Economicus (Smith, 1794) and the Theory of Expected Subjective Profit (Savage, 1954) has influenced the basis on which market research was based until now. It has particularly influenced quantitative research and prediction of future consumer trends (Nicosia, 1966; Engel et al., 1968; Howard and Sheth, 1969).

The economic and rational approach of the human being is left behind (Simon, 1959; Kahneman and Tversky, 1979) revealing that the human being uses heuristics (mental shortcuts) that are far from logical models (León and Botella, 2003). Emotion analysis and cognitive psychology transform the study of consumer behavior and market research (Thaler, 2000; Kahneman, 2011).

It highlights the relevance of emotions in the decision-making process (Zajonc, 1980). Emotion and reason can be understood as two complementary mechanisms in the brain (Mackie and Worth, 1989) when making decisions (Damasio, 1994).

Consumer neuroscience (Escera, 2004) can be defined as the analysis of neural conditions and the processes involved during consumption, as well as the consequences on behavior and its psychological significance (Reimann et al., 2011). Neuromarketing originates from combining neuroscience and marketing, with technological advances that allow us to analyze what brain reactions trigger the stimuli of marketing and communication in consumers (Reimann et al., 2011). The purpose of marketing is to facilitate the meeting between products and people (Ariely and Berns, 2010). Based on this, neuromarketing promotes understanding of the connection of the activity of the neural system with consumer behavior. The theoretical, empirical and practical field of neuromarketing is still developing, due to the fact that it is a young discipline (Garcia and Saad, 2008). Butler (2008) suggests a neuromarketing research model in which marketing professionals, researchers, and other interested parties are interconnected, pointing out that more research is needed to confirm its academic importance (Álvarez del Blanco, 2011).

At present, the new trend of commercialization and marketing must focus its efforts on understanding the behavior and needs of customers and consumers, based on the knowledge of the functioning of the brain that neurosciences applied to the economy provide (Hsu, 2017; Molchanov et al., 2017). The insufficiency of offering quality products or services coexists with the decreasing power of traditional advertising communication, a fragmentation of the media that causes the presence of new channels, especially through the Internet, and the appearance of the informed consumer (Schmitt et al., 2003). Satisfaction currently only guarantees to a certain extent that consumers do not file complaints, but in order to build customer loyalty over the long term, customers, in addition to being satisfied, must be delighted with the brand (Lindstrom, 2008).

It is about the emergence of the culture of experience (Piqueras-Fiszman and Jaeger, 2016). Consumers expect experiences in other environments, such as going to a restaurant or shopping center (not only in traditional places where entertainment is offered as a cinema or a theme park). In this sense, experiences can become complex, personal and provide a lot of information (Manzano, 2011). Sensory marketing (Krishna, 2012) allows brand communication to be managed toward the five senses, with the aim of affecting the perception and purchasing behavior in relation to a product or service (Manzano, 2011) filling the previous deficiencies of a very rational marketing (Daucé and Rieunier, 2002). Create unforgettable experiences, where “sensory pleasure” permeates the client’s consciousness by appealing to the senses (Krishna, 2011). Going beyond the traditional means of sight and sound marketing, brands can establish a stronger and more lasting emotional connection with consumers (Medina, 2008). Multisensory marketing (Filser, 2003) allows the attention of the public (Filser, 2003) to be increased, empowering a rational and emotional client (Schmitt, 2006) who reacts better to emotional and creative stimulation, with stimuli that appeal to senses, feelings and reason itself, with a connection to certain lifestyles with which clients feel identified (Li and Wang, 2017). The techniques and tools used depend on the product being researched (Schmitt, 2006). Available techniques include eye movement analysis, brain focus techniques and traditional verbal methods (surveys, focus group or in-depth interviews).

Neuromarketing studies applied to the food sector aim to provide best practices in food marketing, as well as other aspects such as the perception of nutritional elements (Thomas et al., 2016) the health content of labels (Velasco et al., 2015) the presence of additives and the evaluation of the information transmitted by food packaging (Enax et al., 2015; Hahnel et al., 2015; Fenko et al., 2018; Stasi et al., 2018). The use of eye tracking in order to verify the efficacy of food styling (Tichy et al., 2017) functional magnetic resonance imaging (fMRI) to analyze brain activity against brand logos and their food products, in order to analyze their brain activation (Fehse et al., 2017) or neuromarketing techniques to improve a quantitative study on consumer preferences for organic products (Stoica et al., 2015) are lines of work in research for the food sector. However, everything that is extraordinary (good or bad) becomes ordinary after a time (Smith, 2010). The human mind, in a more or less long period of time, returns to its natural and accustomed state of balance and tranquility (Smith, 2010) regulating the level of emotions, seeking to ensure balance and stability. The brain seeks homeostatic balance (Oatley, 1970) and just as body temperature, blood oxygen, or sleep is regulated (VanItallie, 2006) it also tries to balance the human organism on a psychological level (Wilson et al., 2003) under the concept of emotional evanescence.

Taking this emotional regulation into account, it is important to provide emotional changes that momentarily increase visual retention (peak-valley-peak emotional moments) (Teixeira et al., 2012) this being consistent with a high evaluation of the experience, if it ends with a peak moment of emotion.

### Sample

In the present research, the sample consisted of men and women, according to the profile of the target indicated by the restaurant for the current consumer. A total of 60 people (50% men and 50% women) participated randomly and voluntarily as study subjects after meeting the requirements of being between the ages of 35 and 55 and with a medium-high socioeconomic level. The fieldwork was carried out between January 2018 and February 2019. The location of the study was in the L’Escaleta restaurant, selecting “table zero” for its particular location which was isolated from other diners, measuring subjects of various origins. The study was structured in 2 phases, and in each phase the sample size (consisting of 15 men and 15 women) was suitable for a neuromarketing study (Cuesta-Cambra et al., 2017).

### Data Collection and Analysis

The research phase with the dishes from the tasting menu was carried out using the Pupil Labs manufacturer’s “Pupil Core” eye tracking model, with a sampling frequency of 200 Hz. Pupil Capture software, v.1.23 was used for data collection. The Shimmer3 GSR + model was used to record the electrodermal activity, using the ConsensysPRO software, v.1.6, for data collection. Finally, for recording changes in electrical currents in the form of brain waves, the EEG EPOC + model from the manufacturer Emotiv was used, with 14 channels and saline-based electrodes. EmotivPRO v.2.0 software was used for data collection.

**FIGURE 1:**
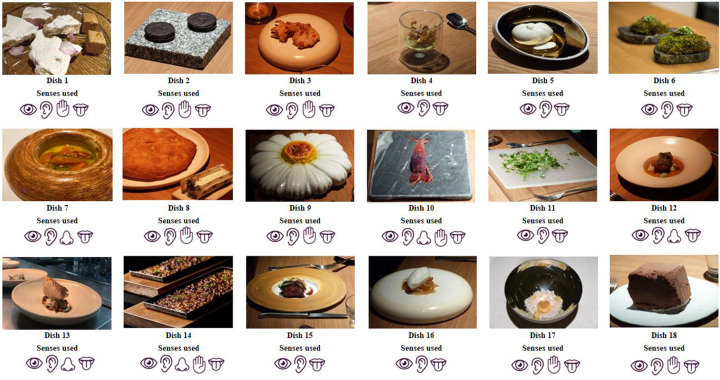
Stimulus (dishes) and senses used. Source: Prepared by the authors.

The statistical analysis of the data was performed with the R software, v.3.6.3. Common elements (stimuli) were defined for all diners (volunteers). The subjects were exposed to a total of 18 dishes, structured into 11 snacks or starters, 4 main courses and 3 desserts (Figure 1 and Table 1). Subjects could not eat while the dishes were presented to them, proceeding to the separation between stimuli – presentation and tasting – to prioritize the areas of interest that captured the most attention (Añaños-Carrasco, 2015).

**TABLE 1:** Stimulus description.

Moment	Dish n°	Description	Abbreviations
Snacks and Starters	Dish 1	Alicante savory nougat/turron	Turrón
	Dish 2	Black and White Garlic Oreo	Oreo
	Dish 3	Chicken cracklings and egg yolk	Chicharrón
	Dish 4	Artichoke hummus	Hummus
	Dish 5	Fresh almond cheese	Queso
	Dish 6	Moss	Musgo
	Dish 7	Pumpkin hedgehogs	Erizos
	Dish 8	Wood sourdough bread with regional herb butter	Pan
	Dish 9	Saffron sabayon with fresh pollen and flowers	Polen
	Dish 10	Salted red shrimp	Gamba
	Dish 11	Wild mustard cream with freshly cut herbs	Mostaza
Main Courses	Dish 12	Blanquet sausage with butterbean and black truffle	Blanquet
	Dish 13	Grilled grouper with mushrooms and hazelnut butter dressing	Mero
	Dish 14	Dry cuttlefish rice with broad beans and artichokes	Arroz
	Dish 15	Strip of roast beef, glazed in its juices with grilled aubergines	Asado
Desserts	Dish 16	Parsnip ice cream with angel hair and muscat vinegar	Chirivía
	Dish 17	In the spirit of a Brioche	Brioche
	Dish 18	Chocolate supermousse with coffee and hazelnut	Supermousse

*Prepared by the authors.*

The independent variables were the age and sex of the participants, with a similar sociocultural profile and determined by the main profile of the restaurant’s clients. The dependent variables were the focus of attention, the peaks of emotional excitement and the levels of brain activity recorded in response to the observed stimuli.

**FIGURE 2:**
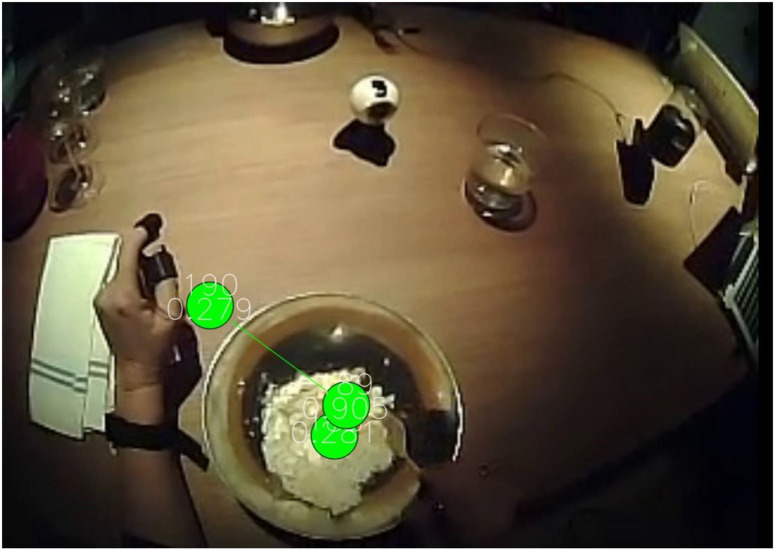
Neuromarketing empirical study. Source: Prepared by the authors.

In the first phase of the study, the dishes were served according to the order established by the chef, collecting the records of eye tracking (visual attention), electrodermal activity (states of emotional excitement) and electroencephalography (interpretation of brain activity based on waves), in order to identify those dishes with the greatest impact on diners (Figure 2).

In order to achieve this regulation of emotions and avoid the saturation and fatigue of diners in a 2 and a half hour experience, we proceeded to a second phase to order the dishes on the menu, interspersing (agreed with the chef) dishes of greater emotional intensity, with less emotionally charged dishes, according to phase 1 records, looking for alternate peak-valley moments, beginning and ending with a peak moment, with the intention of improving the memory of the experience (Renvoisé and Morin, 2007).

Both the dishes and the people who presented them remained constant during the experiment (phases 1 and 2).

Regarding the semi-structured in-depth interview, the interview protocol was designed to provide evidence of the tasting experience. The interviews were carried out by the authors. All interviews were conducted face-to-face. All interviews were videotaped, transcribed, and analyzed.

## Results

### Analysis of the Emotional Intensity of Each Dish (Presentation and Tasting). Phase 1

**FIGURE 3:**
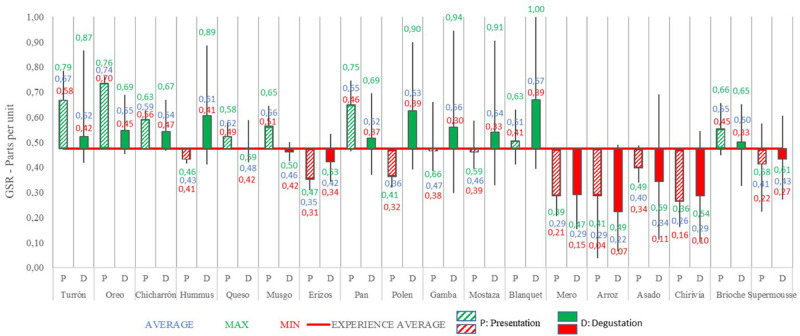
Galvanic skin response analysis of presentation and tasting for each dish: Phase 1. Source: Prepared by the authors.

In the following figure (Figure 3), the emotional level of the presentation phase can be seen, followed by the data from the tasting phase, for each dish:

The average GSR for this phase 1 is 0.47 (parts per unit). The GSR values are referenced with respect to the average gastronomic experience.

Some dishes, such as Hummus, Polen, Gamba or Mostaza (dishes 4, 9, 10 and 11, respectively), show an average of GSR in the presentation below the average, but during the tasting they have risen considerably. The emotional intensity of the tasting was greater than the presentation of the dish.

The dishes whose presentation has shown a higher level of emotional intensity than the tasting have been: Turrón, Oreo, Chicharrón, Queso, Musgo, Pan, Arroz, Asado and Brioche (dishes 1, 2, 3, 5, 6, 8, 14, 15 and 17, respectively).

**FIGURE 4:**
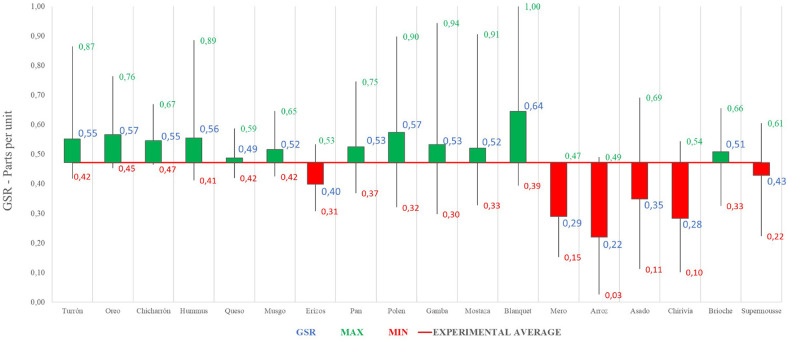
Galvanic skin response joint analysis of presentation and tasting for each dish: Phase 1. Source: Prepared by the authors.

Below (Figure 4) the global value – joint representation – of the emotional intensity of each dish is shown:

The GSR values are referenced with respect to the average gastronomic experience. It should be noted that the dishes with the best record of emotional intensity in Phase 1 have been (in order and above the average value of the experience): Blanquet, Oreo, Polen, Hummus, Turrón, Chicharrón, Pan, Gambón, Musgo, Mostaza Brioche and Queso. Although, the dishes with the lowest register of emotional intensity have been (in order and below average value): Arroz, Mero, Chirivía, Asado, Erizos and Supermousse.

**FIGURE 5:**
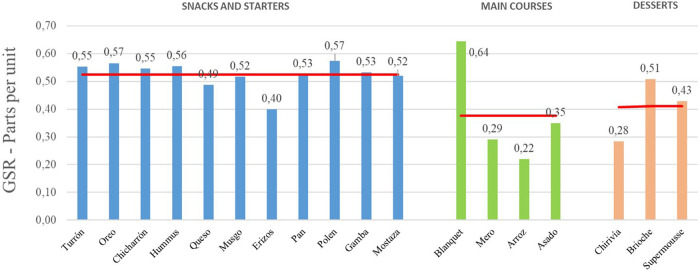
Galvanic skin response joint analysis of presentation and tasting for each dish. Snacks and starters, Main Courses, and Desserts groups. Phase 1. Source: Prepared by the authors.

The following graph (Figure 5) breaks down the level of emotional intensity into three groups, corresponding to starters, main courses and desserts. The average GSR data of the presentation and tasting phases (joint data) of each dish are included. Comparing the levels of emotional intensity by groups of dishes – menu designed by the chef – of the experience, it is observed that there is a lot of difference between these moments, so that the starters are the ones that obtain better ratings (with an average GSR of 0.53), followed by desserts (0.41) and main dishes (0.38).

**FIGURE 6:**
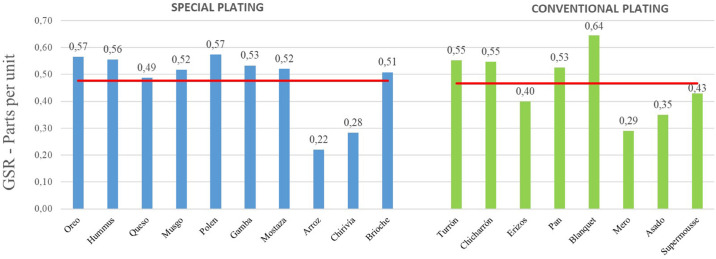
Galvanic skin response joint analysis of presentation and tasting for each dish. Special plating versus conventional plating. Phase 1. Source: Prepared by the authors.

The emotional intensity level registered in the dishes with special plating (the design of the plate is not standard) is then compared with those with a conventional plating (Figure 6). Regarding the dishes that have a special plating, it is worth noting that their average emotional intensity level (0.48) is slightly higher than that of the dishes with a conventional plating (0.47).

**FIGURE 7:**
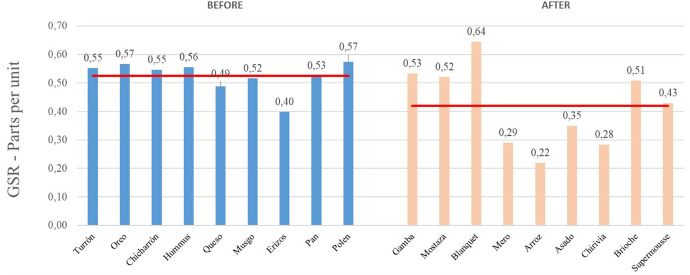
Galvanic skin response joint analysis of presentation and tasting for each dish. Before and after the midway point of the experience. Phase 1. Source: Prepared by the authors.

Regarding the duration of the experience, with an average of 2 h and 32 min, the authors considered analyzing the effect of fatigue on the emotional intensity of the subjects, separating the representation of the level of emotional intensity of the dishes, before and after the midway point of the experience. Figure 7 shows the average GSR data of the presentation and tasting phases of each dish:

As can be seen in the graph, the average emotional intensity level of the first half of the experience is 23,8% higher than that of the second half (0.52 versus 0.42). This raises a possible partial conclusion about the fatigue of the diners, after the midway point of the experience, the moment from which the main dishes and desserts arrive.

**FIGURE 8:**
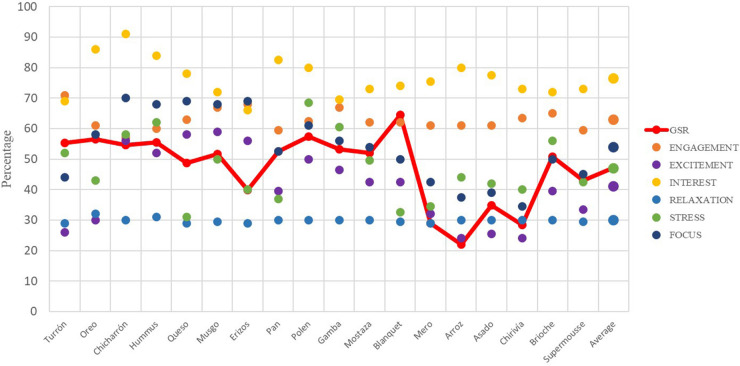
Galvanic skin response and EEG joint analysis of presentation and tasting for each dish. Phase 1. Source: Prepared by the authors.

**FIGURE 9:**
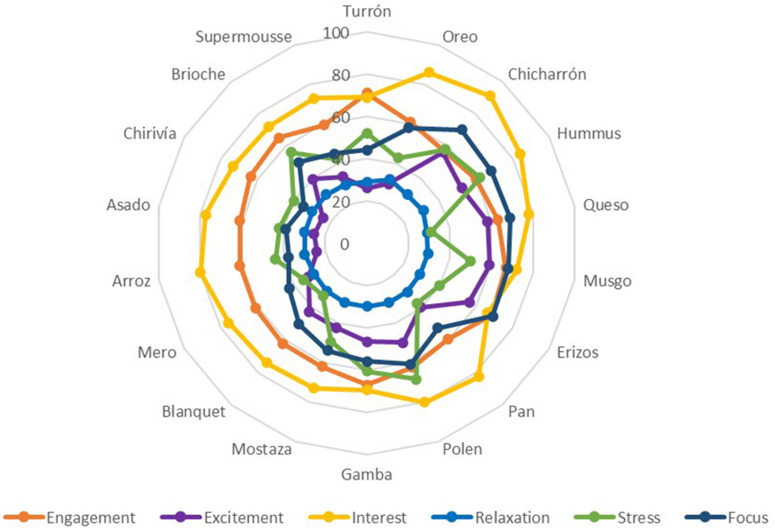
Electroencephalography joint analysis of presentation and tasting for each dish. Phase 1. Source: Prepared by the authors.

The following graphs (Figures 8, 9) show the average percentage of each dish with respect to the GSR and each interpretation value of brain activity obtained using the EEG (Table 2).

**TABLE 2:** Galvanic skin response and EEG recording percentage (Standard error of the mean).

Dish	GSR		Engagement		Excitement		Interest		Relaxation		Stress		Focus	
Turrón	55.2	(1.1)	71.0	(4.7)	26.0	(1.6)	69.0	(4.1)	29.0	(1.2)	52.0	(1.6)	44.0	(0.9)
Oreo	56.6	(5.7)	61.0	(3.6)	30.0	(0.9)	86.0	(2.8)	32.0	(1.6)	43.0	(2.4)	58.0	(1.4)
Chicharrón	54.6	(0.5)	57.0	(2.7)	56.0	(3.6)	91.0	(5.3)	30.0	(1.9)	58.0	(1.7)	70.0	(0.8)
Hummus	55.5	(1.5)	60.0	(1.8)	52.0	(2.4)	84.0	(2.9)	31.0	(0.7)	62.0	(2.4)	68.0	(1.4)
Queso	48.7	(0.4)	63.0	(2.2)	58.0	(2.7)	78.0	(4.6)	29.0	(1.4)	31.0	(1.6)	69.0	(0.6)
Musgo	51.7	(0.6)	67.0	(4.2)	59.0	(1.6)	72.0	(8.5)	29.5	(0.7)	50.0	(4.2)	68.0	(1.4)
Erizos	39.9	(0.6)	68.0	(1.2)	56.0	(3.2)	66.0	(0.8)	29.0	(0.6)	40.0	(0.8)	69.0	(1.7)
Pan	52.6	(0.9)	59.5	(0.7)	39.5	(3.5)	82.5	(2.1)	30.0	(0.2)	37.0	(0.5)	52.5	(4.9)
Polen	57.4	(1.3)	62.5	(0.7)	50.0	(4.2)	80.0	(1.3)	30.0	(0.3)	68.5	(2.3)	61.0	(1.4)
Gamba	53.3	(1.6)	67.0	(5.7)	46.5	(1.2)	69.5	(0.7)	30.0	(2.8)	60.5	(3.5)	56.0	(0.8)
Mostaza	52.1	(1.6)	62.0	(2.7)	42.5	(2.4)	73.0	(4.9)	30.0	(0.8)	49.5	(1.4)	54.0	(1.1)
Blanquet	64.4	(1.7)	62.0	(0.1)	42.5	(2.1)	74.0	(7.1)	29.5	(0.7)	32.5	(2.1)	50.0	(1.4)
Mero	29.0	(0.7)	61.0	(1.4)	32.0	(1.2)	75.5	(1.6)	29.0	(1.4)	34.5	(0.6)	42.5	(0.7)
Arroz	22.0	(1.0)	61.0	(1.4)	24.0	(0.9)	80.0	(1.6)	30.0	(0.1)	44.0	(0.8)	37.5	(0.6)
Asado	34.9	(1.3)	61.0	(1.4)	25.5	(0.4)	77.5	(1.6)	30.0	(0.2)	42.0	(3.9)	39.0	(2.7)
Chirivía	28.3	(1.1)	63.5	(0.7)	24.0	(1.4)	73.0	(0.8)	30.0	(0.1)	40.0	(0.7)	34.5	(3.4)
Brioche	50.8	(0.8)	65.0	(0.1)	39.5	(0.9)	72.0	(0.7)	30.0	(0.1)	56.0	(0.1)	50.0	(5.7)
Supermousse	42.9	(0.9)	59.5	(2.1)	33.5	(3.5)	73.0	(0.8)	29.5	(0.7)	42.5	(0.7)	45.0	(1.4)
Average	47.2	(11.8)	62.8	(3.5)	40.9	(12.4)	76.4	(6.5)	29.9	(0.7)	46.8	(10.9)	53.8	(11.9)

### Analysis of the Emotional Intensity of Each Dish (Presentation and Tasting). Phase 2

Based on the results obtained. identifying emotional intensity as a key aspect for homeostatic balance in emotional regulation, the authors propose a new order – agreed with the chef – that introduces breaks (reductions in emotional intensity) to facilitate the level of perceptual attention, interspersing dishes of higher emotional intensity with dishes of lower emotional intensity, forming a ridge-valley evolution, proceeding to the recording and subsequent analysis of the results in a phase 2 of the empirical part. Table 3 shows the proposed order, ruling out a grouping of dishes based on snacks and starters, and main courses (but keeping desserts for the end of the gastronomic experience):

**TABLE 3:** New order and stimulus description.

Moment	Dish n°	Description	Abbreviations
	Dish 4	Artichoke hummus	Hummus
Mix	Dish 1	Alicante savory nougat/turron	Turrón
	Dish 6	Moss	Musgo
	Dish 5	Fresh almond cheese	Queso
	Dish 3	Chicken cracklings and yolk	Chicharrón
	Dish 7	Pumpkin hedgehogs	Erizos
	Dish 2	Black and White Garlic Oreo	Oreo
	Dish 8	Wood sourdough bread with regional herb butter	Pan
	Dish 10	Salted red shrimp	Gamba
	Dish 15	Strip of roast beef glazed in its juices with grilled aubergines	Asado
	Dish 9	Saffron sabayon with fresh pollen and flowers	Polen
	Dish 14	Dry cuttlefish rice with broad beans and artichokes	Arroz
	Dish 11	Wild mustard cream with freshly cut herbs	Mostaza
	Dish 13	Grilled grouper with mushrooms and hazelnut butter dressing	Mero
	Dish 12	Blanquet sausage with butterbean and black truffle	Blanquet
Desserts	Dish 18	Chocolate supermousse with coffee and hazelnut	Supermousse
	Dish 17	In the spirit of a Brioche	Brioche
	Dish 16	Parsnip ice cream with angel hair and muscat vinegar	Chirivía

*Prepared by the authors.*

**FIGURE 10:**
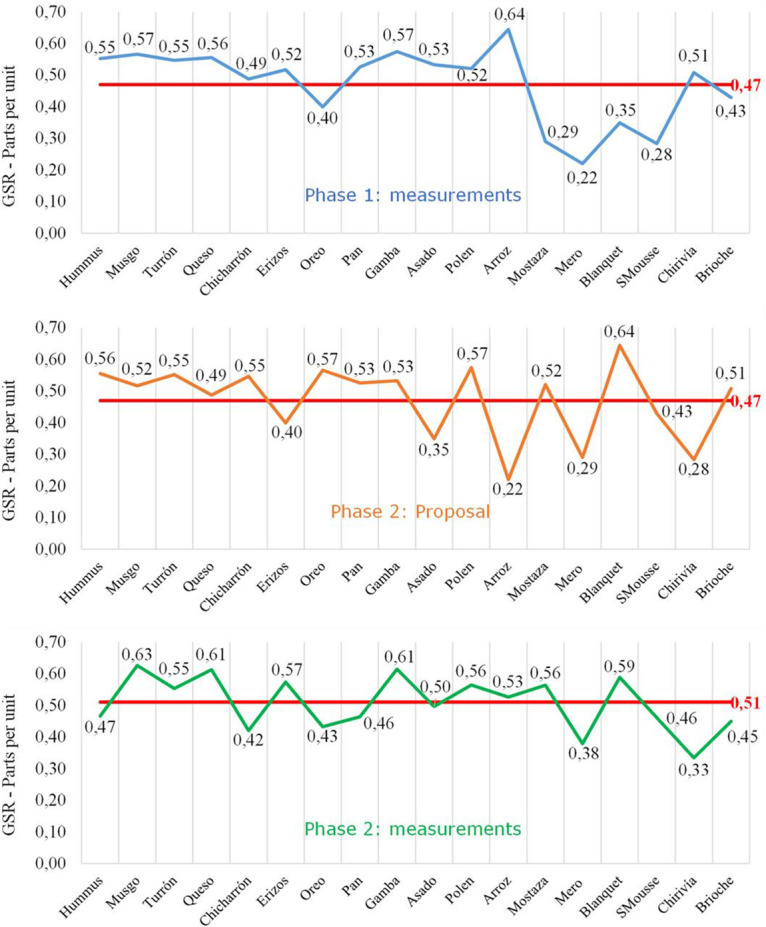
Galvanic skin response joint analysis for each dish in different phases. Source: Prepared by the authors.

**FIGURE 11:**
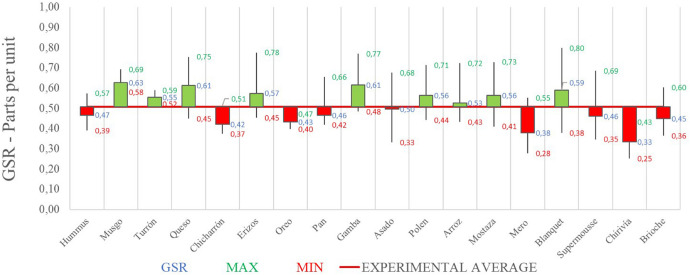
Galvanic skin response joint analysis of presentation and tasting for each dish: Phase 1. Source: Prepared by the authors.

Next, the GSR results obtained in phase 1, the starting proposal for phase 2 (new order of dishes proposed) and the actual result recorded in phase 2 are shown graphically (Figure 10):

In the following graph (Figure 11), the emotional level of the joint experience of presentation and tasting of the phase 2 dishes is represented, whose average emotional intensity value is 0.51.

Thus, the complete experience of the dishes from phase 2 has an average emotional intensity value higher than that of Phase 1, by 8.51% (0.51 vs. 0.47 in phase 1).

Comparing the levels of emotional intensity according to moments of the experience, it is observed that, in this second phase, the first group of dishes (integration of snacks and main courses) obtained a better record (average of 0.53). On the other hand, desserts have registered the same valuation of 0.41 in both phases.

Similarly, there is a greater difference between the average level of emotional intensity of special dishes, compared to conventional dishes (0.52 vs. 0.49), improving the perception of diners of these special dishes in the phase 2 – in the first phase they were 0.48 and 0.47, respectively.

Note that a level of emotional intensity is observed in the first half of the experience (before the midway point) higher than in the second half (0.53 vs. 0.48), but lower than in phase 1 – in the first phase were 0.52 and 0.42, respectively, which may reflect a lower level of fatigue in the second phase of the empirical work (proposal based on the regulation of emotional intensity perceived by the subjects).

### Qualitative Research. In-Depth Interviews

At the end of the biometry part in each phase, an in-depth semi-structured interview was carried out. The questions asked in the interview are related to the dishes tasted, the service, the decoration, the accessories and the overall experience. There was a first phase of open-ended and natural recall questions (about the experience) and a second phase of suggested recall questions.

In both phases the subjects have – in general – a clear memory of the dishes. The experience, in general, was rated by everyone as very good and enriching. The service, the facilities and the food stand out (presentation, ingredients and flavors). Many would repeat the experience. Most have been able to cite about 15 dishes, of the 18 served, and were able to describe them quite well. As for the order in which the dishes were served, they were remembered in groups of starters, main courses and desserts. Among the dishes that really stood out were Blanquet and Mostaza (due to their flavors), as well as Brioche (option to be able to interact).

All diners remember the originality of the plating for some dishes, such as Mostaza, Polen, Gamba, Arroz or dessert dishes, since they represent very well the food on offer.

In the first phase, some subjects highlighted that the experience is a bit long and the main dishes take a long time to arrive, while in the second phase the majority think that the experience seemed very good and that they would not change anything. The order of the dishes is very suitable and they consider it a very complete proposal.

## Discussion

The first part of the study analyses the intersection between consumer behavior, sensory and experiential marketing, and haute cuisine restoration (Cerea and Rurale, 2010), with the aim of designing a guide of recommendations to guide the design of the most appropriate experience strategy, satisfying the needs of haute cuisine and consumers (Olders, 2014). In this area, neuromarketing is very useful as it allows us to efficiently obtain the knowledge, objectivity and precision of the information, as well as the quality of the results (Ohme et al., 2011).

From the biometry application (neuromarketing analysis), it can be highlighted that, according to the galvanic skin response (GSR) records, it is observed that in phase 2 the overall value of the experience is higher than in phase 1 (Figure 10) in 8.51%, a fact that confirms that the order of dishes proposed in phase 2 improves the assessment of the emotional intensity of the experience (Orquin and Mueller, 2013).

The perception of the subjects of the dishes with special plating (Ungureanu et al., 2017) allows a comparison of the level of emotional intensity, because the shape of the dish is more attractive (Bloch, 1995) than in the case of conventional dishes. The same effect occurs in both phases, obtaining an average of 2.12% higher than the emotional intensity level of the dishes with special plating. Furthermore, this difference is even more pronounced in phase 2 (6.12%).

The study of the level of emotional intensity of the first 9 dishes (before the midway point of the experience), comparing them with the following 9 dishes, reveals that the level of emotional intensity decreases over time (Parker and Tavassoli, 2000) to a 23.81%, as well as the interaction with the dishes (Sterner, 1997). The approach of a plate order model based on improving the level of perceptual attention leads to greater activation and emotional intensity in phase 2, after the midway point of the experience, decreasing to 10.42%.

The breakdown of the emotional level into three groups: starters, main dishes and desserts, allows us to observe that in phase 1 there is a range of responses, with the starters obtaining better valuations (GSR average of 0.53, compared to main dishes and desserts, with values of 0.38 and 0.41, respectively). In phase 2, since there was no division between starters and main dishes, it was broken down into 2 groups: starters / main and desserts, with better ratings also being observed in the first block (average value of GSR equal to 0.53). Although with the order of dishes proposed in phase 2, the emotional intensity of the main dishes is increased (compared to phase 1), since the tandem starters plus main dishes of phase 2, obtain the same average as the starters of phase 1. On the other hand, desserts have registered the same evaluation in both phases.

According to the records of brain electrical activity -EEG- (Fakhruzzaman et al., 2015) note that the average of phase 2 is higher than that of phase 1: the values of Engagement, Excitement and Interest are higher in the Phase 2, compared to 1. The stress has the same value in both phases, and the other 2 registers, Relaxation and Focus, are slightly higher in Phase 1. The average EEG values of phase 2 exceed those of the phase 1, both before the midway point of the experience, and after. This also occurs with the GSR values, reflecting the order proposed for the stimuli (Darchambeau, 2005).

Regarding the information obtained from the application of the eye tracking technique (Duchowski, 2007) it is worth commenting that the common denominator in the dishes is that the diner, during the presentation of a dish alternates attention between the Chef and the dish, although in the most striking dishes, the dish is contemplated for a longer time than the Chef. Moreover, during the tasting of the dishes, the diner mainly focuses on looking at the dish he is tasting, especially on those dishes in which the dish must be interacted with.

From the application of traditional marketing (in-depth interview), it can be highlighted that the experience was generally valued by all as very good and enriching in both phases, since satisfied consumers consider their expectations met (Ran et al., 2016). However, consumers are delighted when they have lived an experience that meets their expectations (Martínez-Ruiz et al., 2017). The approach to a sensory experience works with a language capable of activating each of the consumer’s five senses (Lindstrom, 2008). Sensory stimulation positively influences the brand experience and its value, which favorably impacts the purchase intention (Moreira et al., 2017) evoking sensations or feelings associated with the brand (Elder et al., 2011; Mercado, 2018).

Changes in consumer habits make traditional marketing approaches less effective (Wohlfeil and Whelan, 2005). Today’s clients are more independent than ever, and advertising is less effective and more expensive every day (Madinaveitia, 2018). It is important to know how consumers experience brands (Lenderman, 2008) since the differential offer of experiences (Pine et al., 1999) being memorable and personalized, can help protect the company from the erosion of its profit margins and prices, as a result of commoditization of supply and undifferentiation (Pine and Gilmore, 1998; Schmitt et al., 2003).

The limitations considered in this research have been the influence of the restaurant’s name, its geographical location, the chef’s public recognition, the presence of the wine and the sommelier, the influence of repeating the experience or reducing its duration.

## Conclusion

The main objective of this study was the emotional analysis of the experience of tasting a menu of a restaurant with 2 Michelin stars, through neuromarketing, using equipment and knowledge of neuroscience. The methodology performs a global analysis of the tasting experience, using qualitative research techniques and neuromarketing. The analysis takes into account the different aspects of the experience, establishing the variables on which the research is focused, such as eye trajectory, galvanic response of the skin and recording of brain waves.

The greatest contribution of this research has been the design of a new order of the menu of dishes of a tasting experience, based on the metrics of the levels of emotional intensity of the diners. This has allowed us to know the results in the levels of emotional intensity (arousal) of the consumer caused by a proposal to change the order of the dishes, as well as to identify the most highly valued aspects in the consumption of gastronomic experiences, suggested shopping and consumption habits, (understand when the customer would buy a gastronomic experience), the gastronomic projections of the target audience based on the restaurant’s and chef’s brand, and the level of brand memory, product and service, after the high gastronomic experience kitchen.

This research has contributed to the analysis, from the neuromarketing perspective, of the cognitive and emotional options of consumers regarding gastronomic products (Sturiale and Scuderi, 2017). The results can be used to support specific improvement campaigns, through the use of a communication that highlights the emotional components of the purchasing process. Thus, we can speak of a regulation of emotions, referring to processes by which people regulate positive or negative emotions over time, automatically and unconsciously or in a conscious and controlled manner. The purpose of this emotional regulation is to reduce, maintain or intensify the emotional experience resulting from exposure to the stimulus (Gross and Thompson, 2007).

This line of research can be continued with the analysis of the influence of the wine, the duration of the experience, the variation of the emotional impact when repeating it by consumers, or the reversal of the order of the dishes (desserts at the beginning, main dishes in the middle and starters at the end), as proposed by some renowned chefs worldwide. Likewise, contrast the emotional levels of high-quality traditional menus against haute cuisine menus with innovative experiences, or contrast the results of a quantitative investigation with those obtained in the combination of neuromarketing and qualitative research.

Regarding the application of this neuromarketing analysis perspective to other possible areas or sectors, the authors consider that there are key sectors (due to the high emotional component of the product) in which applying this methodology would be very interesting, such as the footwear sectors, clothing, jewelry, luxury vehicles or real estate.

Finally, this study revealed that knowledge of the conscious and unconscious mental states of the consumers allows the design of much more efficient commercial strategies, since it is based on a deeper knowledge of the stimuli that influence the brain and, in turn, consumer behavior and decision making (Morin, 2011). Considering also that consumer habits change, organizations must generate other types of proposals and actions for each contact or communication carried out with their consumers, through all the aspects that accompany the brand, generating a link through the stimulation of the senses and experiences with high emotional charges.

## Data Availability Statement

The raw data supporting the conclusions of this article will be made available by the authors, without undue reservation.

## Ethics Statement

The studies involving human participants were reviewed and approved by Comité de Ética en Investigación – Universitat Politècnica de València. The patients/participants provided their written informed consent to participate in this study.

## Author Contributions

AM-R conceived and designed the experiments, performed the experiments, contributed reagents, materials, analysis tools or data, and wrote the manuscript. VT-V and DJ-V analyzed and interpreted the data, contributed reagents, materials, analysis tools or data, and wrote the manuscript. All authors contributed to the article and approved the submitted version.

## Conflict of Interest

The authors declare that the research was conducted in the absence of any commercial or financial relationships that could be construed as a potential conflict of interest.
